# A Focus on Natural Variation for Abiotic Constraints Response in the Model Species *Arabidopsis thaliana*

**DOI:** 10.3390/ijms10083547

**Published:** 2009-08-13

**Authors:** Valérie Lefebvre, Seifollah Poormohammad Kiani, Mylène Durand-Tardif

**Affiliations:** INRA/IJPB, Genetics and Plant Breeding Laboratory, UR 254, Route de St Cyr, F-78000 Versailles, France; E-Mails: valefebvre@versailles.inra.fr (V.L.); skiani@versailles.inra.fr (S.P.K.)

**Keywords:** Arabidopsis thaliana, natural variation, QTL, abiotic stress, drought, osmotic stress, cold stress, nutrient deficiency, heavy metal stress, light spectrum

## Abstract

Plants are particularly subject to environmental stress, as they cannot move from unfavourable surroundings. As a consequence they have to react *in situ*. In any case, plants have to sense the stress, then the signal has to be transduced to engage the appropriate response. Stress response is effected by regulating genes, by turning on molecular mechanisms to protect the whole organism and its components and/or to repair damage. Reactions vary depending on the type of stress and its intensity, but some are commonly turned on because some responses to different abiotic stresses are shared. In addition, there are multiple ways for plants to respond to environmental stress, depending on the species and life strategy, but also multiple ways within a species depending on plant variety or ecotype. It is regularly accepted that populations of a single species originating from diverse geographic origins and/or that have been subjected to different selective pressure, have evolved retaining the best alleles for completing their life cycle. Therefore, the study of natural variation in response to abiotic stress, can help unravel key genes and alleles for plants to cope with their unfavourable physical and chemical surroundings. This review is focusing on *Arabidopsis thaliana* which has been largely adopted by the global scientific community as a model organism. Also, tools and data that facilitate investigation of natural variation and abiotic stress encountered in the wild are set out. Characterization of accessions, QTLs detection and cloning of alleles responsible for variation are presented.

## 1. Introduction

### 1.1. Arabidopsis

Sequencing the genome of *Arabidopsis thaliana*, the first one completed in plants, was achieved in 2000, bringing new lights on higher plants’ genetics [1]. Its small genome (220 Mb on five chromosomes), its short life cycle (2–3 months in the greenhouse) and its large offspring at each generation (more than 10,000 seeds per plant) have made of this small, mostly self-fertilizing Brassicaceae the model plant in molecular genetics, developmental and cell biology. In the last 15 years, large insertion-mutants collections have facilitated the association of a phenotype with the function of a single disrupted gene. More recently, *Arabidopsis* became an important model species for evolutionary biologists. As almost all selfing species, *Arabidopsis* plants collected in the wild represent nearly homozygous genotypes which are referred to as accessions. Several investigations have been conducted in the past few years assessing pattern of polymorphism, in order to describe the genetic variability of the species. Ultimately, these studies should reveal the demographic event such as population expansion and/or the involvement of selective events that have shaped populations across the world [2–9]. While demographic events and life history have broad consequences on the whole genome; recombination rate, mutation rate and selection have impact on specific loci [9]. Different molecular methods have been used to investigate population structure: Sequencing of short fragments [5,6,8], typing with microsatellite markers [6], SNPs (Single Nucleotide Polymorphism) [6,9] or more recently high density oligonucleotide arrays [2,3]. These findings revealed a strong population structure in *Arabidopsis*, displayed by a deviation from the so called neutral model which is defined by a population of constant size, not subjected to any selective neither demographic event. Whereas previous surveys failed due to the lack of data Nordborg *et al*., using 876 short fragment sequences were one of the firsts to shed some light on population structure in *Arabidopsis thaliana* [5]. To date, most studies suggest that during the Pleistocene, *Arabidopsis* may have restricted to one or several isolated refugia before expanding in a widespread distribution [10]. However, Schmid *et al.* ran a survey on 12 accessions sequenced at 334 loci and concluded that population expansion in *Arabidopsis* is not sufficient to explain the observed patterns of polymorphism [8]. They showed that parameters such as selection should be considered in addition. Recently, a more precise analysis has localized one of the glacial refugia on the Iberian Peninsula [7].

### 1.2. Abiotic Stress

**Figure 1.**
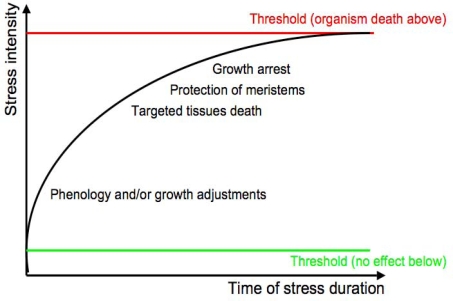
Theoretical graph of the effect of an abiotic stress on plant growth and development.

Plants are particularly exposed to stress from their physical environment, as they cannot move away from disadvantageous surroundings and have to cope with the stress on site. An abiotic stress is due to a non-living environmental factor that can have a negative impact on living organisms. Any given environmental factor beyond its normal range of variation, must adversely affect the physiology, development and/or fitness of the plant and will subsequently be referred to as “stress”. Regarding abiotic stress, a plant has to deal with the type of stress, the combination of different stresses, which effects can be additive or antagonistic, and the intensity and the duration of stress (Figure 1). Development or growth in such adverse conditions can cause extensive losses to agricultural productions [11]. Depending on these physical parameters, the plant will adjust its phenology and/or growth, then will integrate growth arrest, senescence and meristem protection. The perception of the stress signal will determine its transduction and subsequent physiological responses. Along with primary signals, secondary signals such as hormones, Reactive Oxygen Species (ROS) and Ca^2+^ cytosolic fluxes are generated [12].

Constraints such as drought, light and cold are known to induce the hormone abscisic acid (ABA). The regulation of the ABA-responsive genes occurs through *cis-*regulatory sequences such as ABRE (ABA-Responsive Element) or DRE (dehydration-responsive element) present in the promoter [12,13]. The unwanted compounds such as ROS (hydrogen peroxide, hydroxyl radicals, superoxide ions) that plants accumulate in response to an abiotic stress impact the biology of the plant in two ways: either as a secondary signal in different pathways, or through damaging effects in plants due to their cytotoxicity [14]. Therefore their detoxification through the production of antioxydants such as ascorbic acid, glutathione and α-tocopherol may lead to stress tolerance [15]. Indeed, overexpressing ROS scavenging enzymes like SuperOxide Dismutase (SOD), Catalase, Glutathione S Transferase, contributes somehow to stress tolerance [16]. Abiotic stresses also elicit “calcium signatures”, typified by their amplitude, frequency, duration and subcellular localization [17] that could encode specific signals and responses.

Stress response is effective by turning on molecular mechanisms to protect the whole organism and its components and/or to repair damages. For example, damages in plants can be greatly reduced with the accumulation of compatible osmolytes like proline, which is the major one. This accumulation will decrease osmotic potential in the cytosol and facilitate water uptake, but also has other functions such as protecting proteins from misfolding and overcoming the toxic effect of ROS [18]. Reactions vary depending on the type of stress and its intensity, but some mechanisms are commonly turned on because responses to different abiotic stress in plants share common steps, *e.g.,* the osmotic homeostasis is disturbed in drought or in freezing situations by removal of water and in the presence of salt which acts an osmoticum. In addition, there are multiple ways for plants to respond to environmental stress depending on the species and life strategy. Then, it is not appropriate to qualify a genotype as “resistant” to abiotic stress, the term “resistance” being correct in the case of a pathogen attack, where the biotic challenge mediates a gene-for-gene interaction and a qualitative resistance response (vs sensitivity).

Hence, a plant is able to develop in a wide spectrum of soil conditions, with limitations regarding light, temperature, water status, osmotic potential, nutrients, but also toxic metals accumulated in soils as well as the increase of CO_2_ in the atmosphere. Recent studies have revealed many interactions between stress responses. These cross-talks can take place at different levels such as hormonal signalling and reception or transgression of the signals [19].

Many aspects of regulatory mechanisms or signalling pathways may not be revealed through mutants’ studies, partly due to gene redundancy or epistatic interactions. Thus, natural variation and quantitative genetics are promising alternative to detect new functions and genes. The accessions of *Arabidopsis* have each developed adaptive strategies to cope with their potentially deleterious natural environment. It is regularly admitted that populations of a single species originating from diverse geographic origins and/or that have been submitted to different selective pressure, have evolved retaining the best alleles for completing their life cycle. Therefore the study of natural variation in response to abiotic stress, can help to unravel key genes and alleles for plants to cope with their unfavourable physical and chemical surroundings.

**Figure 2.**
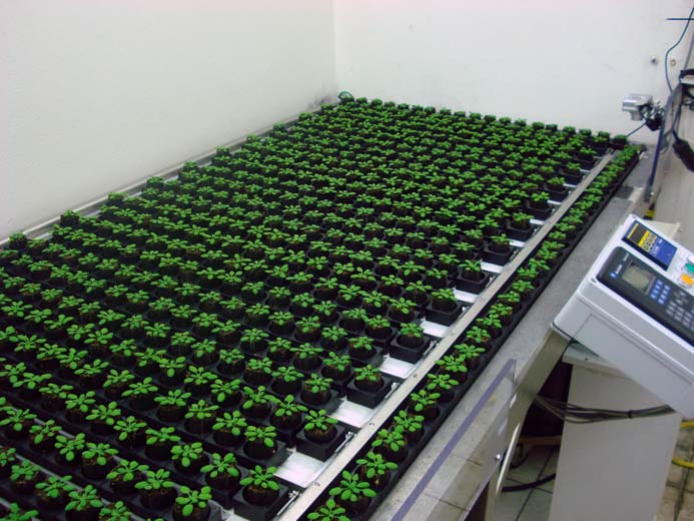
Phenoscope: this *Arabidopsis* culture system created at INRA-IJPB Versailles is able to grow and move 735 plants over the culture table, to adjust their water and nutrient status individually and to monitor their growth by image analysis.

It seems rational to observe the extent of a trait variation before studying its genetic support. Natural variation has been assessed for numerous traits in *Arabidopsis*. This is straightforward for simple characters: as long as the environmental conditions are homogeneous in the experimental system, one can quite easily assess flowering time or biometric parameters. When it comes to stress response one should precisely control stress strength and apply it homogeneously over the experimental system. This requires precise climatic control and homogenization of the physical constraints over the culture space. Greenhouses conditions are quite homogeneous but one cannot reproduce the climatic conditions over the time, which is a problem when studying complex characters. Growth chambers appear as a good alternative to control tightly temperature, light intensity and hygrometry. Even if the quality of the climate control has significantly improved lately, it often remains heterogeneous at the plant scale. Technical solutions have emerged recently to get more and more homogenized culture conditions. Culture robots that continuously move the plants across all positions over a culture space for a given culture cycle are worthwhile as they ensure that all the individual plants perceive the same conditions of light, temperature and humidity on average over the experiment. That type of robots has been set up for crops by private companies (Cropdesign, LemnaTec…). At INRA in Versailles, a so-called Phenoscope devoted to *Arabidopsis* is under proof-of-concept phase (Figure 2).

It is trivial to point out that the results we get of a screen for stress response depend on the traits measured. Therefore, the choice of the trait is crucial. It should be a compromise between an integrative trait that reflects the overall physiological state of the plant (*e.g.,* the rosette surface in *Arabidopsis* [20]) and a trait that reflects a specific aspect of a particular stress response (*e.g.,* the electrolyte leakage, often measured in response to freezing, reflects damages to the membranes [21]). The trait measured is giving one objective insight of the plant response to stress but is not prefiguring stress tolerance “in the field” because of the complexity of the stress themselves and the complexity of the plant response to stress.

### 1.3. Natural Variation

There are two ways to exploit natural variation. One is to seek a common signature among accessions in response to stress, in order to point out a common general mechanism. A large number of characterized accessions coupled with a high density molecular marker map allow the establishment of a correlation between genotype and phenotype. Another is to look for differences among accessions to foresee trait segregation that could be studied in dedicated cross. A continuous range of phenotypes is obtained in segregating populations derived from crosses between two accessions due to different allelic combinations driving the phenotype, combined with environmental effect [22]. Traits that are distributed continuously are referred to as quantitative and the associated genomic regions are called Quantitative Trait Loci (QTLs). QTLs could be analyzed using family-based mapping populations such as F2, Back-Cross (BC) and Recombinant Inbred Lines (RILs), or natural independent populations in Linkage Disequilibrium (LD) mapping or association mapping studies. QTL analysis aims to:

1. Determine the number and position of loci controlling a trait;
2. Estimate the effects and the mode of action of individual QTL;
3. Detect the epistatic interactions among QTLs;
4. Detect QTL by environment interactions (QTLs affecting plasticity);
5. Detect putative pleiotropic effects of QTLs on various traits in the same mapping population.

The main objective of QTL mapping is the identification of sequence change that underlies a specific variant phenotype and the analysis of the causal gene’s responsible for the variation. The identification of a sequence polymorphism that causes natural variation requires laborious cloning approaches [23]. First, a fine mapping is required to reduce the region containing the QTL and subsequently decrease the number of candidate genes. Then, functional genomic strategies would be used to find the precise gene and the nucleotide polymorphisms affecting the function (reviewed in [4,24,25]).

In the present review, abiotic stress commonly encountered by plants in the wild will be reviewed. Each abiotic factor will be characterised, then response variation among *Arabidopsis* accessions will be described and the state-of-art for QTL detection and cloning will be analysed. The involvement of epigenetics variation in response to stress is not included in this review.

## 2. Salt, Drought and Osmotic Potential

Water availability is quite obviously a significant selective agent within natural plant populations. Understanding the genetic and physiological basis of drought adaptation is therefore important for understanding the evolution of wild species as well as for improving crop plants.

*Arabidopsis*, like other annual plants, cope with drought by using three types of adaptive strategies:

1. Drought escape, that allows plant to reproduce and leave an offspring before the environment becomes dry, probably as a life strategy (for review, see Chaves [26]).
2. Dehydration avoidance refers to growth adjustment in order to avoid internal physiological perturbations. Under drought, plants close their stomata to save water and lengthen their roots to reach more water. For most plants, dehydration avoidance is achieved primarily through the regulation of stomatal conductance in order to maintain the internal water status of the plant [27]. However, as stomata are also used in respiration, plants under drought stress need to find a balance in order to maintain a residual carbon assimilation and subsequent growth. This can be measured as the Water Use Efficiency (WUE), the amount of carbon gained per unit water consumed [28].
3. Dehydration tolerance that varies with the genotype is the ability to endure tissue dehydration, sometimes through the accumulation of metabolites, in order to save the whole organism.

These classes are quite theoretical: plants experience such a variety of combined and stochastic stress that responses are complex and mixed.

Natural variation has poorly been exploited to identify new genes or alleles responsible for water deficit tolerance, probably underlying the complexity of this stress. Pigliucci *et al.* studied four accessions, Greenville (GRE), Turk Lane (Tul), Kendalville (Ken) and Landsberg *erecta* (L*er*) in response to different water supplies [29]. Four biometric parameters out of nine showed significant genetic variation among accessions, but no interaction was found, meaning that these accessions react the same way to applied treatments. Meyre *et al.* compared the L*er* and Col accessions as well as their reciprocal F1 progeny, for a series of biometric parameters and ABA content in response to water deprivation [30]. Most of the measured traits were different among the parents and display a dominant inheritance and hybrid vigor. McKay *et al.* did not apply a so called water stress but examined 39 accessions for their WUE [31]. They used the isotopic discrimination against the heavy carbon isotope ^13^C during photosynthesis or “Δ^13^C” to assess the WUE [32]. They observed variation for this trait and a correlation with flowering time. These results confirmed that *Arabidopsis* natural accessions have different strategies to cope with water availability. Nine accessions have been observed for their response to controlled soil water deficit by Granier *et al*. [33]. An-1 shows a noteworthy behaviour, as its final leaf area increases and its transpiration rate per unit of leaf area is maintained with the diminishing soil water content. Bouchabke *et al.* went further, studying four physiological and biometric traits on 24 accessions [34]. They found substantial variation in response to drought: Every trait shows a significant accession × treatment interaction. A Principal Component Analysis revealed that the Cvi-0 and Shahdara accessions are outliers and interesting genotypes to study drought response.

Hausmann *et al.* evaluated the genetic architecture of drought-related traits and their plasticity in response to water stress in L*er* × Col and L*er* × Cvi RILs populations [35]. Flowering time, number of rosette leaves and lateral branches, shoot and root biomass and final number of fruits were studied under well-watered and water-stressed conditions. The majority of traits were controlled by three or fewer QTLs in both populations and some traits such as shoot biomass, fitness and rosette area were influenced by only one QTL in Cvi × L*er* population. For the drought-avoidance mechanism, WUE assessed as Δ^13^C, four QTLs were identified in Cvi × L*er* population and two QTLs were found in Col × L*er* population. L*er* alleles increased WUE as compared to Col and Cvi accessions. The authors reported only two QTLs which control both Δ^13^C and shoot biomass and no QTL were co-located between Δ^13^C and fruit production, which are not genetically correlated in this case. Two trade-off QTLs were observed between Δ^13^C and tissue percent nitrogen as well as between Δ^13^C and branch production indicating the inability of plants to maintain simultaneously high photosynthetic area and high WUE. Interestingly, most traits showed significant phenotypic plasticity as well as QTL × environment interaction and some of the interactions were environmentally dependent. This reveals the complexity of water stress tolerance. In this study, an epistatic interacting QTL was mapped for both branch number and flowering time that had been already detected by Ungerer *et al*. [36]. These results showed that not only QTL location but also some interactions could be consistent across environments.

QTL mapping for flowering time (a drought escape mechanism) and Δ^13^C (a drought-avoidance mechanism) has been also reported in L*er* × Cvi population [37]. Ten genomic regions were observed for these traits and three of the Δ^13^C QTLs detected in this study were previously identified in similar conditions [35]. Interestingly, two Δ^13^C QTLs were located within the genomic position of flowering time QTL which indicated a functional or pleiotropic relationship between these traits. The authors developed Near Isogenic Lines (NILs) containing Cvi and L*er* alleles. NILs segregates at the target locus but identical elsewhere. Cvi alleles at two Δ^13^C QTLs showed increased stomatal conductance and water loss rate, and decreased transpiration efficiency. NILs analysis suggest that genes underlying the observed physiological effects play important roles in controlling stomatal closure during leaf dehydration as well as gas exchange under well-watered conditions.

In other mapping populations, McKay *et al.* analyzed two reciprocal crosses between Kas-1 and Tsu-1 as these accessions are supposedly coming from dry (India) and wet (Japan) areas, respectively, and show extreme opposite WUE [38]. Two genomic QTLs, assessed by Δ^13^C measurement were localized for WUE, the Kas-1 alleles giving a higher WUE. In addition a cytoplasmic component was detected with an inverse effect: Kas-1 cytoplasm giving a lower WUE. This tends to show opposite selective force driving on the nuclear and cytoplasmic compartments for water economy and overall metabolism.

In 2005, Masle *et al.* published the results of a QTL detection for transpiration efficiency (Δ^13^C) in a Col × L*er* mapping population [39]. The causal gene has been identified as the *ERECTA* gene that has also been described for its role in patterning tissues in stems and pedicels. By studying mutants and transformed lines, Masle *et al.* showed that ERECTA is also involved in transpiration efficiency, leaf development and gas exchange in well-watered or in drought environment.

Salt stress, like low temperature is often accompanied by dehydration and osmotic stress. The accompanied low osmotic potential hence reduces water uptake by the plant, resulting in “physiological drought”. The root system shows indeterminate growth and assures two main functions: The anchorage to the soil and the exploration thereof for water and minerals. It is regularly admitted that osmotic potential of the soil has great consequences on the root system architecture. Deak and Malamy have indeed shown a reduction of the root system apparatus when growing plants on osmoticum, due to the repression of lateral root formation [40]. Fitz Gerald *et al.* investigated osmotic stress response of RILs from a L*er* × Col cross, as L*er* has a significantly higher total lateral roots length value than Col on medium supplemented with mannitol [41]. Two QTLs confirmed with NILs were identified: EDG1 and EDG2 (Elicitors of Drought Growth), which have an effect through the regulation of lateral root primordia activity. The Col root system shows weaker performances on osmotic medium, but unexpectedly, the EDG2^Col^ allele promotes the overall root system size.

Salt stress also inhibits plant growth through toxicity, as a result of ion-excess inside the plant [42]. Generally speaking, the acclimation phenomenon allows plants to better endure severe constraints. Hence, plants can become more resistant to salt with a period of acclimation, *i.e.,* an exposure to low salt concentrations [43].

Natural variation for NaCl tolerance was observed among 102 accessions of *Arabidopsis* and the cross L*er*-0 × Col-4 was utilized for mapping the underlying QTLs and candidate genes [44]. These authors showed that NaCl tolerance in this cross is a polygenic trait. Their results also demonstrate that genetic control of NaCl tolerance at germination and vegetative growth stages are independent and they detected five QTLs for each of these traits, both parental lines contributing in positive alleles. The authors found that some of the QTLs identified in their study are mapped close to genes involved in ABA response (ABscisic Acid Insensitive 1 (*ABI1*), *ABI2*, and *ABI3*), ABA biosynthesis (*ABA3*) or ABA modulation (ABA Hypersensitive 1 (*ABH1*)). Some candidate genes involved in other environmental responses such as drought (dehydration-responsive genes *RD26*, *RD29A*, *RD29B*, and Dehydration-Responsive Element Binding Protein *DREB1*) or freezing response (*SFR5*) were also located on the QTLs for NaCl tolerance. Another significant localization was the map position of a QTL involved in the variation in fresh weight. Liu *et al.* showed its co-localization with *SOS2* gene, whose product is a Ser/Thr protein kinase required for salt tolerance during vegetative growth in *Arabidopsis* [45].

Rus *et al.* used natural variation to identify a new *AtHKT1* allele responsible for elevated Na^+^ accumulation in the shoots, which is also associated with NaCl tolerance in *Arabidopsis* [46]. The authors coupled high-throughput elemental profiling with genetics and DNA microarray-based mapping techniques. Analysis of shoot tissue from different *Arabidopsis* accessions revealed that Ts-1 and Tsu-1 collected from geographically distinct populations, respectively coastal regions of Spain and Japan, accumulated higher levels of Na^+^. The cross between each accession (Ts-1 and Tsu-1) and Col-0 showed that high Na^+^ phenotype segregates as a monogenic recessive trait. A candidate gene, *AtHKT1*, which had been previously described as a Na^+^ transporter was found among the two loci identified [47–49]. Complementation tests with a T-DNA insertional null mutant *hkt1-1* establishes that alleles present in Ts-1 and Tsu-1 are responsible for elevated Na^+^ accumulation. An interesting output of this work is that, comparing with a mutant line sensitive to NaCl because of over-accumulation of Na^+^ in shoots, the accumulation of Na^+^ in the shoot of Ts-1 and Tsu-1 does not cause increased sensitivity to NaCl [47,50]. Further analysis of a F2 population derived from Tsu-1 × Col-0 cross showed that the allele of Tsu-1, conferring Na^+^ accumulation, co-segregates with enhanced NaCl tolerance as the homozygous plants for Tsu-1 alleles are able to survive longer in the presence of 100 mM NaCl. The evidence confirmed previous results obtained in *Arabidopsis*: Reduced shoot accumulation of Na^+^ is not necessary for enhanced salinity tolerance [51]. This article showed that two accessions collected from geographically and genetically distinct populations [5] could contain the same *AtHKT1* allele resulting in the accumulation of Na^+^ as well as higher NaCl tolerance. However, it should be mentioned that Ostrowski *et al*. proposed that Japanese accessions might be the result of recent migration/colonization events [6]. The authors proposed an extensive haplotype analyses to identify if this new allele of *AtHKT1* has been under recent directional selection before making any conclusive statements about its adaptive significance.

## 3. Light

Light affects all aspects during development and growth of plant. It is one of the four pathways that are involved in the fundamental developmental switch of flowering initiation (for reviews see Komeda and Roux *et al*. [52,53]). The light-dependent pathway involves the perception and integration of changes in photoperiod, light quality and intensity [54]. The major threat concerning light limitation is shading as it limits significantly the quantity of light perceived by the plant, in particular red and blue wavelengths photosyntheticaly active radiations. In response to crowded population, *Arabidopsis* has developed an adaptive strategy, the shade avoidance syndrome, characterised by elongation of petiols, reduced leaf area and decreased leaf chlorophyll, that would be better designated as “shade escape” as it implies leaving an offspring before the death of the plant [55].

Vascular plants sense light using different photoreceptors specific for different wavelengths: The phytochromes (PHY, red light), cryptochromes (CRY, blue light) and phototropins (UV). Five genes encode phytochrome protein in *Arabidopsis*, named *PHY A* to *E*. They are sensitive to red light (R), far-red light (FR) and more exactly to the R:FR ratio. When subjected to shading, plants are exposed to a low R:FR ratio. PHYA functions as a FR sensor and may be involved in antagonizing shade avoidance. PHYB, PHYD and PHYE act to restrain the shade avoidance phenotypes under high R:FR conditions as *phyB* mutant displays a constitutive shade avoidance phenotype. The other photoreceptors, CRY1, CRY2 and phototropins mediate hypocotyl elongation in response to reduction in blue light quantity.

Another type of light, ultraviolet (UV) radiation, in particular UV-B, can affect the morphology and development of photosynthetic organisms. An excess of UV-B decreases productivity, degrades DNA, induces oxidative stress, leads to proline accumulation, inhibits photosynthesis and reduces stomatal conductance leading to lower carbon assimilation. It has therefore been suggested that higher tolerance to drought stress in *Arabidopsis* plants grown under UV-B radiation could be attributed to the decreased stomatal conductance and increased proline content [56].

The light responses of natural populations and species native of different habitats potentially harbors adaptive variation; therefore understanding the mechanisms underlying photomorphogenic variation is of significant interest [57]. *Arabidopsis thaliana* wild strains grow across a wide range of latitudes, with large differences in light quality, intensity and photoperiod. Response to light has been studied in different accessions. In 1995, Pigliucci *et al*. showed that plain or shaded sunlight generates different response among four accessions, for three traits representing plant growth and size and one trait characteristic of reproductive fitness (number of fruits), out of nine measured traits [29]. High quantity of light applied to L*er* and Cvi accessions, generates a photooxydative stress. The latter can be enhanced with paraquat, an herbicide that causes a light-dependent increase of superoxide radical. In this study Cvi showed a higher rate of stress survival, of chlorophyll and of SOD activity; the higher tolerance of Cvi to light stress is assumed to be caused by an enhanced superoxyde radical detoxification [58].

Light quality received by plants is diverse. Maloof *et al*. studied the response of 141 *Arabidopsis* accessions from around the Northern hemisphere, in five different light conditions: White, blue, red, far-red light and dark [59]. The hypocotyl length was measured as an indicator of response to light. Indeed, after germination the hypocotyl develops until light triggers the transition phase from heterotrophic to phototrophic, stopping hypocotyls elongation, unfolding cotyledons and beginning chloroplasts development. Significant different responses to light were observed and a mild correlation between light response and latitude of origin was detected, in the way that accessions from lower latitudes are less responsive, probably to compensate higher light exposure.

A high FR:R light ratio may be considered as a mimicry of high density plant population and shading because selective radiation absorption by the pigments causes reflection enriched in the FR wave-band. Botto and Smith studied 157 accessions, measuring “days to bolting” and “number of leaves to bolting” as indicators of the precocity of flowering and “hypocotyl elongation”, as indicator of elongation response. The three traits showed extensive variation. The median of the “number of leaves to bolting” is divided by four from white light (no FR signal) to high FR signal suggesting that the response is varying with increased FR light intensity. It is worth mentioning that variation for precocity of flowering and for hypocotyl elongation are not correlated. The authors pointed out candidate accessions for quantitative genetic analysis: Bla-1, Bla-6, Co-4, Ge-2, Lu-1 and Sf-2 because of their unusual weak response to FR [55].

De-etiolation is initiated by red, far-red and blue light and perceived by the plant through photoreceptors (for recent review see Bae and Choi [60]). As the FR pulses induce weak responses, this is a way to explore the genetic control of sensitivity to light. The response of L*er* and Cvi to de-etiolation by FR light pulses are different [61]. Under R pulses only the hypocotyl length witnesses a different response for the two accessions. Magliano *et al.* reported the characterization of 52 *Arabidopsis* accessions in response to FR pulses. They measured hypocotyls length and angle between cotyledons as an indicator of cotyledons unfolding. Most of the accessions show a weak response to FR pulses and the two traits are not correlated. Forty accessions among 52 showed reduced cotyledons unfolding suggesting that this character is commonly repressed in populations [62].

Natural variation regarding response to UV-B has a potential interest in the context of stratospheric ozone reduction which is linked to increased UV-B radiation on Earth. Torabinejad and Caldwell have studied UV-B radiation on seven *Arabidopsis* accessions originating from seven similar environments (100 m above sea level, 3° latitude) [63]. All measured biometric traits showed a genotype effect. A treatment effect as well as a genotype × treatment interaction was observed, except for the shoot architecture parameters. Because the accessions are coming from populations that are not supposed to be exposed to different UV radiation, the results might reflect the intrinsic variation for UV-B tolerance. The mechanisms underlying this tolerance might have other selective functions, like tolerance to other biotic or abiotic stress. Cooley *et al*. studied seven accessions from diverse geographic origin [64]. They observed response to UV-A as well as to combined UV-A + B. Nine biometric traits were measured and three parameters were derived out of these traits. Once subjected to UV-A or UV-A + B, most of the time, inhibitory responses were found. The accessions with the highest growth rate exhibited the highest growth rate inhibition by UV-B + A as illustrated by the linear correlation for these two traits. The only exception is the Aa-0 accession, where most of the traits were unexpectedly promoted by UV-A.

As previously mentioned, plants use different photoreceptors to survey their light environment. Natural variation in light response of *Arabidopsis* has been extensively studied and the causal polymorphism of several light-responsive traits such as seedling emergence or hypocotyls elongation [57,59,65,66] and flowering time [65,67–69] were displayed using natural variation studies.

El-Assal *et al.* described the genetic basis of natural variation for flowering response to photoperiod using QTL map-based cloning [67]. A QTL (named Early Day-length Insensitive, EDI) for flowering response to photoperiod has been identified in a L*er* × Cvi mapping population. Positional cloning followed by transgenic complementation and sequence analysis revealed that the mutation responsible for EDI is a novel allele of *CRY2* which had previously been shown to promote flowering in long day photoperiods [70]. A methionine at position 367 of CRY2 in Cvi instead of valine in L*er*, is the molecular basis of differences in flowering phenotype. This valine residue is highly conserved across plant CRY proteins. The substitution creates a dominant gain-of-function allele which is likely to be sufficient to explain the difference in response to photoperiod between L*er* and Cvi accessions. Interestingly, an exploration of the valine to methionine substitution, among different *Arabidopsis* accessions showed that this allele is very rare and specific to Cvi; its ecological significance being unclear.

Genetic variation between L*er* and Cvi has been widely used for analysis of light responses in *Arabidopsis*. One hundred and five RILs from crosses between L*er* and Cvi were used to investigate the regulation of sensitivity to light signals perceived by phytochromes in etiolated seedlings [61]. Three QTLs were identified for hypocotyl growth inhibition and three QTLs (including VLF6) were identified for cotyledon unfolding under FR light. Fine mapping and transgenic complementation using material developed initially for flowering time QTL showed that the VLF6 QTL is explained by the Cvi allele of *CRY2*, responsible for enhanced cotyledon unfolding under FR pulses. The authors concluded that the blue light photoreceptor CRY2 can modulate seedling photomorphogenesis in the absence of blue light. Interestingly, cytoplasmic effects on seedling de-etiolation were also identified.

QTLs for light response in RILs population generated from Col × Kashmir crosses were identified by measuring seedling hypocotyl length in blue, R, FR, white light and in darkness [71]. From eight QTLs identified, five localized near photoreceptor loci. Two QTLs detected in blue light were associated with *CRY1* and *CRY2*, and two QTLs in R light were near *PHYB* and *PHYC* and finally one QTL in FR was localized near *PHYA*.

It is worth mentioning here that one accession, Lm-2, showed similar responses to light conditions as *phya* mutants, causing reduced FR sensitivity [59]. Sequence comparisons showed that a highly conserved amino acid in the PHYA protein is substituted from methionine to threonine (M548W) in Lm-2 and is responsible for the observed phenotype.

More recently, Balasubramanian *et al*. revealed that a R receptor PHYC controls natural variation for both flowering time and hypocotyl elongation [65]. First, a QTL for flowering time in a Fr-2 × Col-0 population has been identified in the *PHYC* region. A change in the first exon of the *PHYC* gene converting the L299 to a stop codon gives rise to a truncated protein lacking several essential functional domains and is related to the phenotypic variation for flowering time. Results showed that PHYC also accounts for variation in hypocotyl length of 115 wild strains that have been previously investigated by Maloof *et al.* for their response to different light conditions [59]. In addition, PHYC sequence was shown to be more divergent than those of other phytochrome genes in *Arabidopsis*, this gene being potentially under adaptive selection and its contribution to flowering time being latitude-dependent [65].

Filiault *et al*. investigated the involvement of another receptor for R in light sensitivity, PHYB, by sequencing this gene in 140 accessions that have been previously phenotyped for light response of hypocotyls elongation [57,59]. An association mapping and regression analysis revealed one polymorphism as the cause of variation among accessions. Polymorphism in *PHYA* and *CRY2*, as described above, are limited to individual accessions [59,67], making it difficult to know whether they confer adaptation or not to environment. In contrast, alternative variants that cause differential light sensitivity are more common in the case of *PHYC* and *PHYB*, suggesting that they may be important in adaptation [57,65].

The role of hormones in light response has also been studied. DELLA proteins, growth repressors involved in gibberellins signalling, were shown to play a central role in the shade avoidance response, as their degradation increased in canopy-grown plants. This process is suggested to be driven through multiple photoreceptors interplay (for review see [72]). Brassinosteroids are involved in light response as they suppress photomorphogenic growth in the dark [73].

QTLs responsible for natural variation in light and hormone response between the Cvi and L*er* accessions were also identified using 150 RILs [25]. Hypocotyl length was measured in four light environments: White, blue, R, and FR light and in the dark. In addition, white light plus gibberellin (GA) and dark plus brassinazole (BRZ, a brassinosteroid biosynthesis inhibitor) were used to detect hormone effects. Twelve new QTLs, as well as loci harboring known candidate genes were identified. Results showed both environment-nonspecific QTLs and QTL × environment interactions. A major QTL, LIGHT1, maps to an unknown locus with effects in all light environments. In this study, the known *erecta* mutation has been shown to explain the effect of the HYP2 QTL in the blue, BRZ and dark environments, but not in FR. Another QTL, LIGHT2, with effects in white and R light shows interaction with GA and could be the photoreceptor PHYB.

Magliano *et al*. detected a QTL that accounts for the longer hypocotyl in No-0 compared to L*er* under different light or dark conditions in the L*er* × No-0 population [62]. This QTL collocates with HYP2, previously mapped using L*er* × Cvi RILs [61,74]. A QTL that enhanced cotyledon unfolding in L*er* compared to No-0 under continuous FR (UNF1) was also mapped.

More recently Loudet *et al*. described one major QTL (LIGHT5) and its underlying zinc knuckle/PLU3 domain encoding gene (*TZP*) involved in natural variation for hypocotyl elongation response to light [66]. LIGHT5 was detected and fine-mapped on chromosome 5, in Bay-0 × Shahdara derived populations, down to three candidate genes. The authors showed that an 8-bp insertion in *TZP* causes a stop codon in Bay-0, which is sufficient to explain LIGHT5 phenotype. The Shahdara allele increases hypocotyl length. Further genomic analysis showed that this gene is unique and acts downstream of the circadian clock and photoreceptor signalling pathways, regulating blue light-dependent morning-specific growth. However the impact of this gene in adaptation is not yet clear.

## 4. Temperature

The effect of temperature varies extensively, from freezing to hot. Little is known about the molecular basis of the natural variation for freezing tolerance; although cold and freezing conditions are two relevant factors limiting the distribution of plant species. The acclimation phenomenon *i.e.,* exposure to cold or sub-zero temperatures, makes it particular as it conditions the tolerance to freezing temperature [75]. Cold stress results in changing gene expression and membrane lipid composition, accumulation of compatible osmolytes, ABA transient elevation and diminution of plant growth [76]. One example would be the *eskimo1* mutant, described as cold-tolerant by Xin and Browse, which shows an increased proline accumulation [77,78]. Bouchabke-Coussa *et al*. later confirmed that *eskimo1* mutants indeed exhibited higher cold tolerance than the wild type only when exposed to freezing after acclimation [79].

It is admitted that freezing generates an osmotic and a mechanical stress due to ice crystallization. Low temperature also imposes a dehydration stress by lowering water absorption by the root and water transport in the shoot [80]. Indeed, freezing temperature may prevent sufficient water uptake due to progressive solidification of the apoplast which consequently causes reduction of the water potential [13]. Le *et al.* proved that only the sensing of temperature is responsible for sub-zero acclimation as it can be achieved even if water crystallization is avoided [81].

Moreover, Soitamo *et al.* shed light into the necessity of light for induction of some cold responsive genes, in particular the *CBF*s. CBFs or C-repeat Binding Factor also called DREB1 for Dehydration-Responsive Element Binding Factor are transcription factors induced by cold and water stresses. They also showed an increase in the production of ROS and the up-regulation of certain genes encoding ROS scavenging proteins under these conditions [82].

Le *et al*. characterized four accessions for their cold and freezing acclimation capacity measuring electrolyte leakage and deducing a Leakage Temperature 50 (LT_50_): A temperature where 50% of electrolyte leakage occurred [81]. They have shown a great variation in the sub-zero acclimation depending on the temperature and the time of exposure. There is no correlation between cold and freezing acclimation capacity suggesting their genetically independent basis. This was in accordance with another study showing that the Col accession is more freezing tolerant than C24, with or without acclimation [83]. Hasdai *et al.* showed that observing growth parameters on accessions at 6 °C and 14 °C could not be used to predict tolerance to freezing [84]. Zhen and Ungerer observed considerable differences in freezing tolerance among 71 accessions that showed a clinal pattern with latitude of origin, with or without acclimation [85]. The authors suggest that inducible cold response has a cost which might be counterselected in geographical zone that are not likely to experience freezing stress. Additional experiments are needed to confirm this hypothesis. McKhann *et al*. also show important variation for tolerance to -5 °C exposure after acclimation [86]. They reinforced the fact that freezing tolerance is a complex character showing that *CBF* and *COR* (COld Regulated) genes respond differently to stress among eight accessions, though there is no clear correlation between gene expression, sequence polymorphism and freezing tolerance. Thereby, freezing tolerance is complex and *CBF* genes by themselves cannot explain the phenotypic variation. Global transcript and metabolite profiles have been observed by Hannah *et al*. on nine accessions subjected to freezing with or without acclimation [87]. They observed considerable variation and a significant correlation between freezing tolerance and accessions’ habitat of origin. They also demonstrated that accessions that are able to acclimate showed a 1.5- to 2-fold changes in global gene expression compared to non-acclimated plants. Moreover, no clear relationship has been found between metabolite changes and acclimation capacity. Swindell *et al.* studied gene expression patterns in response to cold (4 °C) and to heat (38 °C). About 70% of the genes are responding to stress but only 43 of them are considered as adaptive, *i.e.,* their expression level co-vary with the average temperatures in the geographic origins of the accessions [88]. Another study relates the effect of chronic low-temperature on biometric parameters and gene expression among 23 accessions [89]. Variation is observed for root elongation rate at ambient and at low temperature but except for Er-0, all the accessions respond the same way.

Several studies have been conducted to assess the flowering time in response to ambient temperature variations, like 16 °C and 25 °C [65,90,91], which do not constitute a stress *per se* as only two stress responsive genes were induced at 25 °C. Results showed that a slight temperature variation can induce dramatic effect on flowering time and variation among accessions. An important part of the thermal adjustment is not due to a typical heat shock, vernalization or photoperiodic response, but might reflect a complex genetic network.

Analysis of natural variation leaded to identify *CBF2* as a candidate gene for freezing tolerance. Alonso-Blanco *et al*. identified QTLs controlling freezing tolerance under long day and short day photoperiods: Seven different loci on chromosomes 1, 4, and 5, which were named FREEZING TOLERANCE QTL 1 (FTQ1) to 7, were detected. In all of them, the L*er* allele increased freezing tolerance compared to Cvi [92]. Only two QTLs, FTQ4 and FTQ6, were detected in both photoperiod conditions, suggesting that freezing tolerance is also photoperiod-dependent. FTQ4, which showed the larger effect, maps to the region containing a tandem repeat of three highly similar *CBF* genes (*CBF1*, *CBF2* and *CBF3*). By sequence analysis the authors showed that the 1.6-kb Cvi deletion in *CBF2* promoter region including the *ICEr1* (Induction of CBF Expression region 1) sequence and probably other regulatory elements might be the cause of the low cold induction [93]. However, *CBF3* also shows a large amount of polymorphisms between L*er* and Cvi. The involvement of several genes in FTQ4 is somehow supported by the fact that the three *CBF* genes do not operate independently [94].

## 5. Mineral Nutrition

Plants must acquire all the mineral nutrients they require for survival, including trace elements, from the complex environment of the soil. Ionomics is a developing functional genomic strategy designed to rapidly identify the genes and gene networks involved in regulating how plants acquire and accumulate these mineral nutrients from the soil [46]. Lahner *et al*. screened 6,000 fast-neutron-mutagenized *Arabidopsis* plants grown under unstressed conditions, and identified 51 mutants with altered shoot elemental profiles [95]. They estimated that 2% to 4% of the *Arabidopsis* genome is involved in regulating the elemental composition or ‘ionomics’ [96]. Potentially, all kind of nutrient depletion constitutes an abiotic stress. It is supposed for long that response to nutrient depletion is linked to root plasticity as the larger the root surface is, the better soil exploration would be for nutrients uptake, especially for phosphorus which is an immobile and poorly accessible compound [97].

Phosphorus (P) and nitrogen (N) are the two major nutrients for plant growth [98]. In response to phosphate starvation, which happens notably in acid soils, *Arabidopsis* plants tend to improve their uptake while increasing root apparatus size. *Arabidopsis* plants adapt their root architecture in order to improve their uptake: The primary root growth stops and numerous new lateral roots emerge (Reviewed by Raghothama [99]). In addition, numerous root hairs appear, their length being inversely correlated with the P concentration in the medium [100]. It has long been shown that the phytohormone auxin and the growth regulator ethylene are involved in the modulation of the root architecture via altering primary root growth, promoting root hair and lateral root formation [101]. More recently, the joint action of auxin and ethylene have been related to the modulation of root apparatus in relation to the amount of P in soil [102]. In acid soils, aluminium and iron will induce P deficiency, due to the formation of insoluble complexes unavailable for plant uptake. On the contrary, in alkaline soils, P bounds magnesium or calcium and forms weakly soluble compounds [103]. In order to cope with P-limiting conditions, plants increase excretion of organic acids like citrate, whose high affinity for cations will displace P from their insoluble complexes, making it available for plant uptake [104].

*Arabidopsis* has also developed adaptive responses to N-limited soils: Increased root growth, photosynthesis reduction, N remobilization from older organs leading to senescence and accumulation of protective compounds like anthocyanins. However, the mechanisms of uptake and acclimation to these stress are less documented [105,106].

In 2000, Narang *et al.* deeply analyzed five accessions for their root morphology and phosphate uptake parameters in response to phosphate depletion [107]. Three of them (C24, Co and Cal) showed a high Phosphate Acquisition Efficiency (PAE), the other two (Col-0 and Te), showed a lower PAE. Meanwhile the efficient accessions display a higher root hair surface on phosphate-free medium. It is tempting to speculate that the two characters are linked. More recently, exploiting a QTL approach, Reymond *et al.* studied six accessions for their Primary Root Length (PRL) and total lateral root length [108]. The root architecture is already different under standard growth condition and the response to phosphate starvation is even more dramatic, *e.g.,* PRL reduction is of 75% in Shahdara accession and of approximately 20% in Bay-0 and L*er*. An N:P:K (nitrogen:phosphorus:potassium) gradient has been applied to four accessions resulting in a genotype × treatment interaction on five out of nine studied traits [29]. Low nutrient has drastic effect on plant traits, ending with smaller plants and lower vegetative growth [109]. A complementary study obtained with 26 accessions under low and high nutrient status, shows a genotype × treatment interaction, most of the variation being due to a limited number of accessions with highly distinctive phenotypes, the other ones responding similarly to low nutrient treatment [110].

Plants acquire N from the soil mainly in the form of nitrate. Nitrate limitation has several direct and indirect consequences on growth through modifications of nitrate availability in plant cells and tissues or on osmotic pressure [111]. Loudet *et al*., presented the genetic analysis of water and anion content variation in the Bay-0 × Shahdara population of RILs in response to nitrate availability [112]. Water, nitrate, chloride, and phosphate contents were measured at the vegetative stage in the shoots of plants grown in two N conditions. A total of 34 QTLs were observed for the studied traits. Interestingly, two well-known flowering-time loci, FRI and FLC, were shown to be likely involved in the control of water content variation independently of N availability. The authors also localized two major QTLs for chloride and phosphate content in limiting N conditions implemented map-based cloning and showed that they both map on chromosome 1.

In another publication, Loudet *et al.* used the same mapping population with the aim of dissecting the N metabolism pathway in *Arabidopsis*. They measured shoot growth, total N, nitrate and free amino acids contents in high or low nitrate availability conditions. These traits were significantly correlated in both environments. They identified 48 QTLs in total, mapping at 18 loci at least, most of them being strongly affected by N limitation in soil. Four to nine QTLs per trait have been found and from none to five QTL × QTL epistatic interactions, which therefore seem to play an important role in controlling phenotypes. Both positive and negative allelic effects were found. Four loci can be retained as source of major variation in one or both N environments, which co-localized or not with known genes involved in N metabolism, which demonstrates once again the power of this approach to uncover new genes and pathways [113].

The effect of phosphate deficiency on root development has been studied and a major QTL (LPR1) involved in primary root growth arrest in response to low phosphate and in the control of primary root cell length has been identified in the RILs population derived from the Bay0 × Shahdara cross [108]. The underlying gene, which encodes a MultiCopper Oxidase (MCO) highlights the essential role of MCOs in plant development [114]. LPR1 is differentially expressed in the root cap leading to the modification of the activity and/or the distribution of a hormone-like compound that triggers the primary root development from indeterminate to determinate growth, which consequently reduces cell elongation and promotes lateral roots formation [115].

Sulfur (S) uptake is essential for the biosynthesis of sulfured amino acids, cell metabolism, and stress responses. A decrease in S uptake can always be linked to many metabolic modifications that strongly change the growth of the plant. Moreover, S transport and metabolism as been linked to different hormone pathways such as auxin, cytokinins and jasmonic acid [98].

A gene involved in natural variation for sulphate content in relation with N availability has been described by Loudet *et al.* [116]. Using a RILs population from the Bay-0 × Shahdara cross and candidate gene approaches, the authors showed that a gene encoding an adenosine 5’-phosphosulfate reductase (*APR2*) is involved in sulphate content variation between Bay-0 and Shahdara accessions. An alanine residue changed to glutamate was found to be responsible for sulphate accumulation in the Shahdara accession, because of a reduced APR activity. Interestingly, the Shahdara allelic effect was stronger when N availability was limitant in soil, confirming the tight relationship between sulphate assimilation and N metabolism, as an interesting explanation for the QTL × environment interaction.

Boron (B) is another essential microelement for plant growth and development. B deficiency has been reported to decrease yield in many crop plants [117]. QTLs for B efficiency or tolerance to low B stress, were identified in a 101 RILs population from a L*er* × Col cross, in two separate experiments [118]. Then two RILs that were genotypically and phenotypically different were crossed to further confirm one major QTL, AtBE1-2, as a monogenic locus in the F2 population. The B efficiency coefficient and seed yield under low B conditions were investigated: Five and three QTLs were identified respectively for each trait. Interestingly, some QTLs are common between the two traits. An effort for searching candidate genes showed that ten B-related genes, together with a B transporter homolog (*BOR5*) were located in the QTL region of AtBE1-2.

Molybdenum (Mo) is also an essential element for living organisms, which, in the form of molybdopterin-cofactor, participates in the active site of enzymes involved in key reactions of carbon, N, and S metabolism [119]. It is notably involved in nitrate assimilation, sulphate detoxification, ABA biosynthesis and purine degradation [120]. Low pH is a major soil constraint to agricultural production, reducing yield on nearly 25% of the world’s land presently under production and the availability of Mo in acid soils is drastically reduced so that Mo deficiency is a widespread agricultural problem [121,122]. For soils above pH 4.23 and those rich in organic matter, the soluble oxyanion, molybdate (MoO_4_^2−^) becomes the predominant available form [123].

Natural variation has been used in *Arabidopsis* for understanding the genetic basis of Mo content in plants. Baxter *et al.* studied natural variation for whole plant Mo content, coupled with QTL based genomic approaches to identify the causal genes [124]. Using a RILs population from the cross L*er* × Col-0, the QTL and its underlying gene *(MOT1*) has been identified. The authors showed that a 53 bp deletion in promoter of *MOT1* is strongly associated with low *MOT1* expression and low shoot Mo content in L*er*-0, Van-0 and across 92 other accessions. *MOT1* belongs to the sulphate transporter superfamily and can specifically transport Mo. It is required for efficient uptake and translocation of Mo, as well as for normal growth under limited Mo supply. This study is an example of how natural variation has been useful to identify a Mo transporter, localized in the mitochondrion, that controls both root and shoot Mo content in *Arabidopsis*. At the same time, Tomatsu *et al*. discovered and functionally characterized *MOT1* in a L*er*-0 × Col-0 RILs population [125]. These studies provided the first molecular insight into the processes that regulate Mo accumulation in plants.

## 6. Metals

Heavy metals, such as cadmium (Cd), selenium, mercury, etc… are micronutrients essential for plant growth, which can become toxic when present in excess in soil (reviewed by Clemens [126]). Their concentrations in plants are related to their solubility in the soil. It is assumed that only metal ions enter the plant cells and elicit responses to toxicity. These opportunistic ions use cation channels and transporters of low specificity such as those for Zn^2+^, Fe^2+^ and Ca^2+^. The major site of heavy metal sequestration is the vacuole of root cells, via binding of the ions to glutathione (GSH) or GSH-derived peptide. Else, detoxification may occur from the excretion of organic acids, such as citrate or malate, out of the root tip and their subsequent chelation of metal ions, as it is the most common tolerance mechanism for aluminum [127,128]. The symptoms generated by toxic metal ions uptake are mainly side effects. An example would be the water balance disturbance, resulting from the inhibition of stomatal opening caused by metal stress and the subsequent proline accumulation. After Cd^2+^ exposure, plants present the symptoms of an oxidative stress: This phenomenon is probably due to the binding of Cd^2+^ to GSH, a ROS scavenging compound (reviewed by Schützendübel and Polle [129]). Noteworthy, the chemical similarity of toxic ions such as Cd^2+^ with Ca^2+^ or Zn^2+^ has strong detrimental consequences on signalling cascades [126].

Genetic analysis of Cd tolerance has been also reported in *Arabidopsis* interspecific cross [130]. *A. halleri* (Cd-tolerant accession) was used to map QTLs and to identify putative candidate genes underlying Cd tolerance in a first-generation backcross progeny from the cross between *A. halleri* ssp. *halleri* (pollen donor) and its non-tolerant relative *A. lyrata* ssp. *petraea*. The *A. halleri* genotype was shown to be tolerant to Cd concentrations and able to hyperaccumulate Cd. 196 individuals of the A*. halleri* × *A. lyrata* ssp. *petraea* backcross population (BC1) was used for QTL mapping and three QTLs on chromosomes 3, 4, and 6 were detected. At all three QTLs, the *A. halleri* allele had a positive effect on Cd tolerance. The heavy metal transporter gene ATPase4 (*HMA4*) colocalized with the major QTL and was consequently considered as a candidate gene potentially responsible for the phenotype. Results showed that elevated expression of *HMA4* is likely the mechanism for improving Cd/Zn tolerance in plants under conditions of Cd/Zn excess by maintaining low cellular Cd^2+^ and Zn^2+^ concentrations in the cytoplasm.

Rhizotoxicity of aluminum (Al) in acid soil is a major environmental stress [131]. At pH below 5.5, Al^3+^ cations are solubilized in the soil solution from aluminosilicate complexes to levels that inhibit root growth and function [122,131]. The stunted, poorly functioning root systems that result from Al toxicity directly reduce plant vigour and yield, increasing susceptibility to other stress such as drought and nutrient deficiency. According to Taylor, plants have two types of Al tolerant mechanisms, namely “internal Al tolerance” and “Al exclusion” [132]. Internal Al tolerance is associated with detoxification of Al in the symplast, such as chelation with organic acids or scavenging Al-induced superoxides through the action of GSH S-transferase [133,134]. On the other hand, Al exclusion is associated with detoxification of Al in the rhizosphere by increasing the pH of the rhizosphere [135]. Some plants are excreting organic acids to modify the pH at the root surface [136]. For example, Al-activated root malate release has been reported as an Al tolerance mechanism for *Arabidopsis* and wheat, and citrate release has been reported for maize Al tolerance [127]. This suggests that higher plants possess a series of Al-tolerant mechanisms in which many Al-tolerant genes would be involved.

Al^3+^ and H^+^ stresses are linked in field conditions. Ikka *et al.* studied relative root length on Al^3+^ and pH 4.7 (excess of H^+^) hydroponics media relative to standard culture medium, among 260 accessions [137]. They succeeded in differentiating Al^3+^, H^+^ and Al^3+^ + H^+^ combination tolerant accessions (*e.g.,* Lö-2 and Co-4). However no correlation has been observed between Al^3+^ and low pH tolerance. Their results indicate that different genetic mechanisms are involved in response to Al^3+^ and H^+^.

QTL and epistasis for *Arabidopsis* Al tolerance were analyzed using a RILs population derived from a cross between L*er* and Col [138]. Al tolerance was defined as relative root length of plants grown on 4 μM AlCl_3_. Two main QTLs and five independent epistatic interactions were detected. Interestingly, key enzymes related to enhanced organic acid excretion were adjacent to the epistasic loci identified between chromosome 1 and chromosome 3 [139]. Another Al-tolerant gene with an increasing rhizosphere pH was mapped on chromosome 4, where an epistatic interaction of the relative root length was detected with chromosome 3. Also, an Al sensitive gene was mapped on the chromosome 5, where an epistatic interaction occurs [140,141]. Overexpression of a blue copper-binding protein resulted in enhanced Al tolerance in L*er* and a homologue of this protein collocate with an epistatic locus on chromosome 2 [142]. However, a fine-scale mapping is necessary to identify whether or not the epistatic loci and the Al-tolerant genes are identical.

Hoekenga *et al.* identified two significant QTLs (QTL1 and QTL2) in a L*er* × Col RILs population for Al tolerance estimated as root growth [127]. One of them located on chromosome 1 (QTL1) corresponds to QTL identified previously by Kobayashi and Koyama [138]. These two major QTLs apparently act together in the same pathway, based on evidence for epistasis, and together explain 40% of the variation in Al tolerance among the RILs. Candidate gene approaches showed that seven Al-inducible genes are located at the QTL1 and eight mapped at QTL2. Thirteen of the fifteen genes have nucleotide differences within the predicted coding sequences or in closely flanking regions in L*er* accessions, which may contribute to differences in expression or function between L*er* and Col. On the basis of their annotation, three of these genes may encode proteins involved in organic acid transport or metabolism. A malate transporter gene, *AtALMT1* (Al-activated Malate Transporter), has also been identified as critical for Al tolerance in *Arabidopsis* using forward and reverse genetic approaches [143]. However, the location of *AtALMT1* on chromosome 1 is not consistent with the major QTLs detected for Al tolerance on the same chromosome in this study. These results suggest that other factors are involved in Al tolerance and underline the complexity of this trait.

## 7. Carbon Dioxide

As autotrophic living organisms, plants build their biomass from atmospheric carbon. Actual gradual elevation of atmospheric CO_2_ (0,550 mg/g predicted in 2050 [144]) results in higher growth rate and yield. Indeed, the stomatal aperture is reduced leading to lower evapotranspiration in C3 and C4 species. This is part of an acclimation phenomenon. In addition, photosynthesis is enhanced in C3 plants due to an increase in the carboxylation rate mediated by the key enzyme Rubisco (substrate-limited at current CO_2_ concentration) and a reduction of photorespiration (for review, see Ainsworth and Rogers [145]). Elevated CO_2_ concentration also leads to a decrease in Rubisco quantity in leaves [146] and an increase in the expression of genes related to ROS metabolism and chaperone [147].

In 1995, Zhang and Lechowicz analyzed two accessions, Cvi-0 and Ms-0, in response to CO_2_ elevation (≈ 700 ppm), at low or high nutrient supply [148]. Among 16 traits, the only significant difference in response to CO_2_ between the two accessions was observed for the rate of the rosette diameter growth. The results showed that the different sensitivity to nutrient supply does not imply a different sensitivity to CO_2_. Li *et al.* used the Free Air Carbon dioxide Enrichment system (FACE) that allows plants grown in the field to be subjected to CO_2_ increase (≈ 550 ppm) [149]. They tested three accessions and analyzed the metabolite and the transcript profile responses. Among genes varying between ambient and elevated CO_2_, a large number are involved in metabolic functions. The authors were seeking for a signature across the accessions that could reflect a generalized response to elevated CO_2_. All signature genes that harbor an annotation have already been reported in response to abiotic stress, according to the hypothesis of Miyazaki that elevated CO_2_ constitutes a stress *per se* [150]. Tonsor and Schneider observed 35 accessions under four CO_2_ concentrations from 250 ppm, corresponding to pre-industrial atmosphere to 710 ppm which is the highest projection for the XXI^st^ century. A large panel of traits have been measured along plant life-time witnessing growth and phenology, in an attempt to build an explicative model for the observations. The outputs of this study are large. Changes in carbon supply result in plastic positive response on most traits measured, across the gradient. There is a significant “accession effect” on trait variation measured after bolting, consequently a possible effect on fitness, but this study focused on variation across treatments to feed a model. There is a stronger global effect of the lowest CO_2_ concentration (250 ppm) than the highest (710 ppm), as if the use of excess CO_2_ was strongly constrained by other parameters, probably water and N uptake rates [151].

Cross *et al*. performed a study among 24 accessions under short days, low light and N excess conditions that are limiting for carbon assimilation and which mimics growth under carbon limiting condition [152]. This is not a stress response study but a good illustration of the great amount of information that can be obtained by studying natural variation under monitored abiotic situations. The authors analyzed the rosette weight as an integrative trait, as well as metabolites and seven enzyme profiles. Results are showing both general features across the accessions like the significant positive correlation between rosette fresh weight and enzyme activities (highest score for aspartate aminotransferase) and divergent strategies carried by some accessions (*e.g.,* the low weight and high enzyme activities of Pa-1). The seven enzyme activities involved in central metabolism are positively correlated. There is no overall trend of rosette biomass and metabolites concentration.

## 8. Other Abiotic Stress

Flooding, waterlodging or even heavy rainfalls saturate the soil with water and induce anoxia that affects root system. One study has been performed with 47 accessions under flooded or non-flooded treatments [153]. Among the nine traits measured, three showed a significant accession × treatment interaction including root fresh weight.

Oxidative stress can be mimicked with the application of atrazine, a xenobiotic that fixes to the photosystem II, consequently blocking electron transfer and generating ROS. Ramel *et al.* tested 11 accessions in response to atrazine (manuscript in preparation). They observed large variation in atrazine sensitivity, assessed with biometric and metabolic traits and in ROS accumulation. The authors established correlations between the response to oxidative stress and the endogenous level of soluble sugars.

## 9. Conclusions

This review constitutes an attempt to survey the large and promising possibilities allowed by the study of natural variation and its genetic basis. There is obviously variation in natural population for a range of traits reflecting adaptation to local abiotic environment that is accessible to experimentation with all the necessary care. All these studies report the behaviour of different natural accessions in designed controlled experimental systems, figuring abiotic stress (see Table 1). Thanks to the international effort dedicated to *Arabidopsis thaliana*, QTL mapping and confirmation is accessible routinely in research laboratories. As listed in Table 2, many QTL related to diverse abiotic constraints have been cloned, using different genetic approaches. Different factors that could greatly contribute to the achievement of QTL detection are listed bolow:

1. The mapping population should be large enough to maximize the recombination events,
2. The studied trait should be easy to screen and show variation among the population lines,
3. Molecular markers should be sufficiently dense on the genome, particularly in the region of interest.

For subsequent cloning, the QTL should explain a substantial proportion of the trait variation to allow its detection in segregating NILs.

Genomic resources like accessions, RILs and sometimes NILs are now available to the global scientific community, like at the Genomic Resource Centre at INRA Versailles (http://dbsgap.versailles.inra.fr/vnat/). The great power of natural variability is also illustrated by the fact that natural alleles can be used to confirm the implication of a gene in a trait (*e.g.,* the complementation of *apr-2* mutant with natural allele driving shoot sulfate content [116]. The success story of the natural variation studies in *Arabidopsis* is due to the implementation of interdisciplinarity between population and quantitative genetics, eco-physiology and all the “omics” techniques at the genome scale. No doubt that in the near future, natural variation studies will keep growing our knowledge of integrated plant response to environmental stress. However, in spite of the increasing interest for quantitative genetics, the discovering of new genes’ function can not be split from classical genetics. Mutants’ approaches and alleles’ complementations will always be the key steps to conclude for the gene underlying a QTL.

Another available tool that comes from human genetics, might be very useful in the near future to identify genes involved in the variation of quantitative traits. Genome-wide association study or Linkage Desequilibrium (LD) mapping (for reviews see Buckler and Thornsberry and Yu and Buckler [154,155] aims at unravelling the genetic determinism of complex characters making direct use of natural populations, thus exploring the largest genetic basis available in the species. This tool does not need any dedicated segregating population as QTL mapping does. Beside the availability of a population of accessions, one need to genotype and to establish the structure of this population to avoid spurious associations caused by eventual mating restrictions and other effects of population size and dynamics [156]. In *Arabidopsis*, globally, the linkage disequilibrium is not too extended: Markers around most gene loci rapidly become unlinked. Consequently, the method needs a rather high–but accessible-density genotyping. Until now, the tool was not yet applied to map loci involved in abiotic, but in biotic stress response [157].

As a final point and to stimulate reflection, Fu’s *et al*. brief communication is mentioned: the authors integrated transcript, protein, metabolic and phenotypic data to detect QTLs for 40580 molecular and 139 phenotypic traits [158]. They show that only six loci (5cM window) are merging from 16% to 77% of the transcript to the phenotypic variation. This analysis tends to show that, for a given RILs population, major genes are pleiotropic and/or are buffering overall genome variability. This important communication should be taken into account, keeping in mind the huge number of possible cross to scramble together parental genomes and the chance that loci might cover several causal genes.

## Figures and Tables

**Table 1.** List of studies reporting characterization of *Arabidopsis* accessions in response to abiotic stress.

**Accessions 1**	**Stress**	**Trait**	**Reference**
	**Light, Radiations**		

4	100, 80, 60 and 40% of sun light	Biometric and fitness parameters	[29,109]
157	Far-red light (3 red/far-red rates)	Flowering time, hypocotyl length	[55]
141	White, blue, red, far-red and dark	Hypocotyl length	[59]
52	Far-red pulses	Hypocotyl length, angle between cotyledons	[62]
7	UV-B	Biometric traits on vegetative (2) and reproductive (4) apparatus.	[63]
7	UV-A, UV-B + A	9 biometric traits on vegetative (6), reproductive (2) and root apparatus (1) and 3 derived parameters.	[64]

	**Temperature**		

21	15, 20 and 25 °C	Flowering time (day of flowering, total leaf number at flowering), height at flowering	[91]
9	Cold and freezing acclimation	Electrolyte leakage, LT_50_, genes and metabolites expression	[87]
4	Cold and freezing acclimation	Electrolyte leakage, LT_50_, leaf sugar content	[81]
71	Cold acclimation, sub-zero temperatures	Tissue damage index	[85]
50	Freezing tolerance (with acclimation)	Tissue damage index, *CBF* and *COR* genes expression	[86]
12	6 °C, 14 °C and freezing	five biometric traits, flowering time, electrolyte leakage, anthocyanin and chlorophyll contents	[84]
10	Cold (4 °C), heat (38 °C)	Gene expression	[88]
150	16 °C	Flowering time (day of flowering, total leaf number at flowering)	[90]
52	25 °C	Flowering time (total leaf number at flowering)	[65]
23	10 °C chronic exposure	Root elongation rate, candidate genes expression	[89]

	**Nutrient**		

4 36	0, 2, 4 or 6 N:P:K fertilizer doses	Biometric and fitness parameters	[29,109,110]
36, then 5 contrasted	Phosphate depletion (hydroxylapatite or 2,5 μM KH_2_PO_4_)	Root morphology parameters, phosphate uptake kinetics	[107]
6	Phosphate depletion (5 μM NaH_2_PO_4_)	Root morphology parameters	[108]

	**CO_2_**		

3	CO_2_ “enrichment” (550 ppm)	Gene expression, metabolite profile	[149]
35	CO_2_ low, standard and enriched (250 to 710 ppm)	Biometric, developmental and metabolic parameters	[151]
24	Reduce CO_2_ assimilation (Short days, low light, excess nitrate)	Rosette weight, enzymes and metabolites profile	[152]

	**Root anoxie**		

47	Soil saturated with water (waterlogging)	Biometric parameters	[153]

	**Heavy metal**		

260	Al^3+^ 1 mM or pH 4.7	Relative root length	[137]

	**Oxydative**		

11	Atrazine (0.25, 0.5 μM)	Metabolic and root morphology parameters, ROS accumulation	Ramel *et al*., in prep.

	**Osmotic (including drought, salt)**		

4	5, 10, 15 or 20 mL of water in 2 inches pot	Biometric and fitness parameters	[29]
39	No stress	WUE (delta^13^C)	[31]
9	Monitored drought stress	Projected leaves area, Transpiration rate	[33]
24	Monitored mild drought stress	Total Leaf Area, Relative Water Content, Electrolyte Leackage, Cut Rosette Water Loss	[34]
102	250 mM NaCl *in vitro*	Germination, Fresh weight, dry weight	[44]
12	100 mM NaCl in pots	Survival	[46]

^1^ Only the studies that comprise more than two accessions are reported in this table.

**Table 2.** Summary of the characteristics of the QTLs cloned, for abiotic environment-responsive traits in *Arabidopsis*.

**Trait**	**QTL**	**Gene**	**Function**	**Approach**	**aR^2^%**	**bPlant number**	**cResolution (kb)**	**dCandidate gene and early or late evidence**	**Identification of QTN**	**Population**	**Functional proof**	**Reference**
Photoperiod responsive flowering time	*ED1*	*CRY2*	Cryptochrome	Positional cloning	28–56	1822	45	Yes (late)	Amino acid substitution	L*er* × Cvi	Transgenic complementation	[67]
Light responsive flowering time and hypocotyls elongation	-	*PHYC*	Phytochrome	Positional cloning	-	140	1000	Yes (early)	Amino acid substitution	Fr-2 × Col-0	Quantative complementation	[65]
Light responsive hypocotyls elongation	*LIGHT*2	*PHYB*	Phytochrome	Association mapping	18–22	140 accessio ns	NA	Yes (early)	Amino acid substitution	L*er* × Cvi	Transformation	[57]
Light responsive hypocotyls elongation	*LIGHT5*	*TZP*	Zinc Knucke	Positional cloning	40	4600	7	Yes (late)	Premature stop codon	Bay-0 × Sha	Transgenic complementation	[66]
Sulfate content	SOC.1	APR2	5’-Phosphosulfate reductase	Positional cloning	48	411	4000	Yes (early)	Amino acid substitution	Bay-0 × Sha	Transgenic and quantitative complementation	[116]
Molybdate content	-	MOT1	Mo transporter	Positional cloning	-	18	172	Yes (early)	53-bp deletion in promoter	L*er* × Col-0	Heterologous system (yeast)	[125]
Molybdate content	-	MOT1	Mo transporter	BSA-Positional cloning	-	200	346	Yes (early)	53- bp deletion in promoter	L*er* × Col-0	Quantitative complementation	[124]
Na^+^ accumulation	-	AtHK T1	Na^+^ transporter	BSA-Positional cloning	-	60	2000	Yes (early)	Deletion in upstream of AtHKT1	Ts-1, Tsu-1 × Col-0	Quantitative complementation	[46]

^a^ Percentage of phenotypic variance explained by QTL;

^b^ Number of plant screened;

^c^ Map resolution;

^d^ Along the fine mapping process.

## Abbreviations:

ABA: Abscisic Acid
Al: Aluminum
BC: Back-Cross
BRZ: Brassinazole
BSA: Bulk-Segregant Analysis
bZIP: Basic region/leucine ZIPper
CBF: C-repeat Binding Factor
Cd: Cadmium
CDPK: Calcium-Dependant Protein Kinase
Col: Columbia
COR: COld Regulated
CRY: Cryptochrom
DRE: Dehydration-Responsive Element
FACE: Free Air Carbon dioxide Enrichment
FR: Far-Red light
GA: Gibberellin
GSH: Glutathione
HKT: High affinity K+ Transporter
K: Potassium
LD: Linkage Disequilibrium
N: Nitrogen
NIL: Nearly Isogenic Line
P: Phosphorous
PAE: Phosphate Acquisition Efficiency
PHY: Phytochrom
QTL: Quantitative Trait Locus
QTN: Quantitative Trait Nucleotide
R: Red light
RIL: Recombinant Inbred Line
ROS: Reactive Oxygen Species
SNP: Single Nucleotide Polymorphism
SOD: Super Oxyde Dismutase
UV: Ultraviolet
WUE: Water Use Efficency
